# Tfh Cells in Henoch‐Schönlein Purpura (IgA Vasculitis): Current Concept

**DOI:** 10.1155/jimr/4143288

**Published:** 2026-05-28

**Authors:** Junke Huang, Juan Liu, Yue Hu, Xuan Zhang, Qing Zhang

**Affiliations:** ^1^ Department of Dermatology, Second Xiangya Hospital, Central South University, Hunan Key Laboratory of Medical Epigenomics, Changsha, China, csu.edu.cn; ^2^ Department of Dermatology, The First Affiliated Hospital of Hainan Medical College, Haikou, Hainan, 570105, China

**Keywords:** B cell, Henoch-Schönlein purpura, IL-4, IL-6, T follicular helper cell

## Abstract

Henoch‐Schönlein purpura (HSP) is an inflammatory condition affecting the small blood vessels, leading to organ damage and symptoms across various body systems, including skin, joints, and kidneys. The disease’s pathogenesis involves the deposition of immunoglobulin (Ig) A immune complexes in vessels, triggering inflammation and damage. Recent research highlights the role of T follicular helper cells (Tfh cells) in HSP. The review delves into Tfh cells’ biological functions, emphasizing their involvement in the HSP’s development by activating autoreactive B cells and inducing the production of autoantibodies through the secretion of interleukin (IL)‐21, IL‐4, and IL‐6. Additionally, the roles of different Tfh cell subsets in the pathogenesis of HSP are also characterized by their unique features. We also highlight the relationship between Tfh cells and galactose‐deficient IgA1 (Gd‐IgA1) production, compare Tfh‐mediated immune responses in HSP with those in IgA nephropathy (IgAN) and systemic lupus erythematosus (SLE), and discuss emerging therapeutic strategies targeting the Tfh–B‐cell axis. It concludes by suggesting the need for further research on Tfh cells to understand HSP pathogenesis better and develop effective treatments.

## 1. Introduction

Henoch‐Schönlein purpura (HSP), also known as immunoglobulin (Ig) A vasculitis, is an inflammatory illness that causes inflammation, tissue damage, and organ involvement in the body’s tiny blood vessels. It is most common in children and young adults [1, 2]. The major pathogenic feature of HSP is the deposition of immunological complexes comprising IgA in tiny blood vessels, which causes a range of symptoms that might affect the skin, joints, gastrointestinal system, kidneys, lungs, brain, and other organs [3]. Although HSP can be self‐limiting to some extent, some patients may have recurrent episodes, especially those with HSP nephritis (HSPN) [4]. In severe cases, the disease can progress to chronic renal failure, severely affecting the patient’s standard of life [5]. Although the specific origin of HSP is unknown, it is thought to be a multifactorial illness resulting from a complex combination of environmental, gene‐related, immunological, and lifestyle variables [6]. These intricate interactions possess the capacity to incite immune system activation, culminating in the genesis of IgA immune complexes. These complexes can then deposit in the small blood vessels, triggering an inflammatory response and leading to tissue damage [7]. In recent years, an increasing amount of data suggests that immunological factors, particularly T follicular helper cells (Tfh cells), perform an essential part in the pathogenesis of HSP.

Tfh cells, as an assortment of CD4^+^ T lymphocytes, play an important role in B‐cell activation. Through interactions with B cells, Tfh cells can support the formation of long‐lasting plasma cells and memory B cells, leading to long‐term protection against infections [8]. In this review, we will concentrate on the involvement of Tfh cells in HSP pathogenesis to gain an understanding of the processes of HSP formation and progression.

## 2. Primary Anatomical Sites and Molecular Mechanisms of Tfh Cell Differentiation

The differentiation of Tfh cells is regulated by a complex transcriptional program. B‐cell lymphoma 6 (Bcl‐6) is considered the master transcription factor controlling Tfh cell differentiation and germinal center (GC) formation. CD4^+^ T cells lacking Bcl‐6 fail to differentiate into Tfh cells. By interacting with transcription factors such as retinoid‐related orphan receptor‐γt (RORγt) and T‐bet, Bcl‐6 promotes the expression of C‐X‐C chemokine receptor type 5 (CXCR5), programmed cell death protein‐1 (PD‐1), and other Tfh‐associated molecules while suppressing differentiation toward other CD4^+^ T‐cell lineages, including T helper 1 (Th1) cells, T helper 2 (Th2) cells, and regulatory T cells [9]. In addition, costimulatory signals such as inducible costimulator (ICOS)–ICOS ligand (ICOSL) interactions and cytokine signaling pathways including interleukin (IL)‐6, IL‐21, and signal transducer and activator of transcription 3 (STAT3) further promote the differentiation and maintenance of the Tfh lineage [10].

Tfh cells primarily differentiate within secondary lymphoid organs, including lymph nodes, the spleen, and mucosa‐associated lymphoid tissues (MALTs), where GC reactions occur [8]. In IgA‐mediated diseases such as HSP, mucosal immune tissues—including the tonsils and gut‐associated lymphoid tissue (GALT)—are considered particularly important sites for IgA immune responses. These tissues provide specialized microenvironments that facilitate interactions between antigen‐presenting cells, T cells, and B cells, thereby supporting GC formation and promoting Tfh cell differentiation and B‐cell maturation [11].

Importantly, a proportion of Tfh cells generated within GCs can exit lymphoid tissues and enter the peripheral circulation as circulating Tfh (cTfh) cells, which retain the ability to provide help to B cells and are considered surrogate markers of ongoing GC activity [8, 10]. Increased frequencies of cTfh cells have been reported in patients with HSP and may reflect enhanced Tfh differentiation within secondary lymphoid organs during disease development [12].

Unlike other subsets of CD4^+^ T lymphocytes, Tfh cells preferentially migrate to lymphoid follicles and exhibit a unique ability to express multiple activating or costimulatory molecules simultaneously. These molecules include ICOS, CXCR5, PD‐1, IL‐21 receptor (IL‐21R), IL‐6 receptor (IL‐6R), and CD40 ligand (CD40L), all of which play important roles in antibody production by B cells [8]. Among these molecules, ICOS, CXCR5, PD‐1, and CD40L are regarded as characteristic surface markers of Tfh cells and are commonly used to identify this subset [13]. The expression of CXCR5 allows Tfh cells to migrate toward B‐cell follicles along the chemokine gradient of C‐X‐C motif ligand 13 (CXCL13), facilitating their interaction with B cells within GCs [14].

## 3. Biological Functions of Tfh in Autoimmunity

Tfh cells were shown to play a crucial part in triggering the production of autoreactive B lymphocytes in autoimmune reactions. Tfh overexpression promotes the activation, differentiation, and proliferation of autoreactive B lymphocytes via the release of factors like IL‐21 and IL‐4, along with IL‐6, resulting in the production of autoantibodies. These autoantibodies can form immune complexes with self‐antigens, which deposit in tissues, subsequently leading to chronic inflammation and tissue damage. This process is considered a key factor in the progression of numerous autoimmune illnesses, like HSP, lupus, rheumatoid arthritis (RA), etc. [10, 15–17].

IL‐21 represents the primary effector cytokine secreted by Tfh cells. It has been proven that it plays critical roles in GC development, B‐cell proliferation and differentiation, as well as Ig class switching [18, 19]. It also plays an essential part during the initial immune response of B lymphocytes to T lymphocyte–dependent antigens and secondary immune responses, as well as the continual upkeep of humoral immune responses [20]. IL‐21 not only induces differentiation of all B cell groups, involving naïve B lymphocytes, transitional B lymphocytes, GC B lymphocytes, and memory B lymphocytes, into Ig‐secreting cells that secrete large amounts of IgM, IgG, and IgA but also has regulatory effects on Tfh cells via autocrine signaling, further promoting GC formation [21, 22]. Additionally, IL‐21 can activate STAT3, causing naïve T cells to differentiate into Tfh cells [23].

Another effector cytokine secreted by Tfh cells, IL‐4, is a pivotal molecule in immune regulation, exerting regulatory roles in various immune cells and responses [24]. IL‐4 can boost B‐cell activation and proliferation; induce IgG1, IgE, and sIgM production; influence the amount of IgE Fc receptors on B lymphocytes along with monocytes; and play a role within the development of numerous allergic illnesses, including eczema and asthma [24, 25]. IL‐4 has also been demonstrated to stimulate the development of naïve CD4^+^ T lymphocytes into Th2 cells, which aid in the proliferation, differentiation, and antibody generation by B cells [26].

Tfh cells also play a role in triggering B cells and Ig class switching via IL‐6 secretion [14]. IL‐6 is an immunoregulatory protein regarded as a multifunctional cytokine, influencing immune cell expansion, differentiation, and revitalization [27]. When the body’s inflammatory mechanisms are activated, IL‐6 can induce the production of acute‐phase proteins, leading to leukocytosis, fever, and vascular generation, thereby amplifying acute inflammatory responses and promoting autoimmunity [28]. The function of IL‐6 in the immune response as well as other physiological processes is complex and diverse, and its abnormal expression or overactivation is associated with various diseases, for instance, RA, systemic sclerosis, and experimental autoimmune uveoretinitis [29].

CXCR3 and C–C chemokine receptor 6 (CCR6) were used to phenotype four subpopulations of CD4^+^ CXCR5^+^ Tfh cells. These subpopulations include CXCR3^+^ CCR6^‒^ Tfh1 cells, CXCR3^‒^ CCR6^‒^ Tfh2 cells, CXCR3^‒^ CCR6^+^ Tfh17 cells, and CXCR3^+^ CCR6^+^ Tfh1/17 cells [30, 31]. In humans, IL‐12 facilitates the proliferation of Tfh1 cells by activating STAT1 and STAT4 signals [32]. On the other hand, the IL‐4‐STAT6 signal drives Tfh2 cell differentiation, while the TGF‐β‐STAT3/STAT4 signal prompts Tfh17 cell differentiation [33]. Although the function of Tfh1 cells in autoimmunity is unknown, Tfh2 and Tfh17 cells were identified as having pathogenic functions in many autoimmune illnesses, such as RA, systemic lupus erythematosus (SLE), and idiopathic inflammatory myopathies [34].

Studies indicate that Tfh1 cells are induced during immune responses to a variety of infections, involving HIV, influenza, and malaria [35]. Tfh1 cells can express Th1‐related factors CXCR3 and T‐bet. Furthermore, under the presence of IL‐2, IL‐4, and IL‐12, Tfh1 cells can generate IL‐21 and interferon‐γ (IFN‐γ) [36, 37]. Notably, Tfh1 cells play a crucial part in the development of T‐bet–expressing B lymphocytes by secreting IFN‐γ and IL‐21 [35]. T‐bet^+^ memory B cells are now recognized as a separate group of effector/memory B lymphocytes that arise from infections, age, and autoimmune and play an important role in humoral immunity [35]. Additionally, IL‐21 and IFN‐γ have the ability of eliciting class‐switching of GC B cells, thereby promoting the production of IgG1 [38]. Tfh2 cells can express the Th2‐associated factor GATA3 and increase the generation of IgG4 and IgE antibodies via IL‐4 and IL‐21 production [34]. This mechanism assumes a critical role of Tfh2 cells in IgE‐mediated allergic diseases [38–40]. Tfh17 cells, on the other hand, express the Th17‐related factor RORt as well as generate IL‐17 and IL‐22, primarily inducing the production of IgA [12]. These Tfh17 cells exhibit a long‐lived memory cell phenotype and are recognized as one of the long‐lived antigen‐specific memory Tfh cell subsets [41]. Importantly, Tfh17 cells stand out as highly effective inducers of antibody responses, conferring advantages in immune memory maintenance, persistence, and support for antigen restimulation, ultimately leading to potential long‐term protection [41, 42].

In summary, diverse types of Tfh cells perform distinct roles within the body. Tfh1 cells hold significance in humoral immunity, yet their precise involvement in autoimmune diseases remains enigmatic. Conversely, Tfh2 cells promote the production of IgE and IgG4 antibodies, closely linked to allergic diseases. Meanwhile, Tfh17 cells excel in generating long‐term immune protection. These cells exert their influence on immune responses by secreting various cytokines that support B‐cell proliferation, divergence, and class switching.

## 4. Roles of Tfh Cells in IgA Vasculitis

### 4.1. Tfh Cell Subsets

Liu et al. [43] investigated the roles of different groups of Tfh cells in HSP pathogenesis. The findings revealed an increase in the number of circulating Tfh2 and Tfh17 cells among every HSP kind, with a positive connection between the two. Serum IgA levels were shown to be favorably related to the amount of both of these cell types. Tfh2 and Tfh17 cells are able to assist naïve B cells by generating IL‐21, which then secrete diverse Ig isotypes. This could represent one of the explanations of high blood IgA levels among HSP patients. Furthermore, in HSP patients, the amount of Tfh2 cells has been shown to be favorably connected with IL‐4 levels and inversely correlated with blood complement 4 (C4) levels [43]. Tfh2 cell quantities that are abnormally high could boost IL‐4 production, resulting in a stronger inflammatory response and worsening of C4 exhaustion, culminating in its accumulation onto the wall of the vessel; all of these are connected with the development of HSP. In conclusion, these findings indicate that Tfh2 and Tfh17 cells have a role in the pathophysiology and progression of HSP. In individuals with abdominal HSP, the amount of circulating Tfh1 cells was shown to be lower [43]. This phenomenon may be related to the expression of CXCR3 on their surface, which can cause Tfh1 cells to migrate to inflammatory organs, resulting in a decline in the amount of circulating Tfh1 cells [43]. These suggest that in abdominal HSP, Tfh1 cells may migrate to the gastrointestinal tract and participate in inflammatory reactions, thereby leading to the occurrence of gastrointestinal symptoms [43].

### 4.2. B Lymphocytes

Tfh cells play a crucial role in the development of HSP because of their capacity to promote B‐cell activation, proliferation, and differentiation, thereby contributing to autoantibody production. Several studies have found a large increase in B‐cell counts in individuals with HSPN, particularly activated B cells. The B cell count corresponds with clinical measures such as 24‐h urine protein in patients, indicating that B lymphocytes play an important role in the etiology of HSPN [44]. The efficacy of B‐lymphocyte depletion therapy utilizing anti‐CD20 antibodies in the treatment of HSP highlights the essential function of B lymphocytes in the pathogenesis of this illness [45].

One of the central pathogenic mechanisms in HSP is the increased production of galactose‐deficient IgA1 (Gd‐IgA1), which represents a key component of the widely accepted “multi‐hit hypothesis” explaining IgA‐mediated disease pathogenesis [46, 47]. In this model, B cells produce aberrantly glycosylated IgA1 molecules that are subsequently recognized by antiglycan autoantibodies, leading to the formation of circulating immune complexes that deposit in tissues and trigger inflammatory responses [46]. Gd‐IgA1 levels in HSP patients’ blood have been found to be elevated [14]. Compared to normal IgA, Gd‐IgA1 is more prone to self‐aggregation, leading to the formation of large molecular substances and a heightened tendency to create circulating immune complexes [48]. Simultaneously, when the spatial structure of Gd‐IgA1 changes, it becomes more difficult to identify and remove, resulting to an increase in circulating Gd‐IgA1 and associated immunological complexes [49]. Gd‐IgA1 circulating immunological complexes readily deposit inside the mesangial region and underneath the endothelium of renal glomeruli, activating the complement pathway. They also promote the growth and stimulation of mesangial cells as well as other inflammatory cells, leading to the production of inflammatory substances, fibrogenic factors, and chemotactic factors, causing an increase in the mesangial matrix [50]. Furthermore, Gd‐IgA1 has the ability to increase the reactivity of nitric oxide (NO) synthase while decreasing the activity of vascular endothelial growth factor and decreasing blood vessel reparative capacity. Ultimately, these processes lead to the occurrence of kidney damage, including glomerulosclerosis and interstitial fibrosis [49, 51–53].

Emerging evidence suggests that Tfh cells may contribute to Gd‐IgA1 overproduction through multiple interconnected mechanisms. Tfh cells secrete large amounts of IL‐21, a key cytokine that promotes B‐cell proliferation, plasma‐cell differentiation, and Ig class switching [10]. In addition, Tfh‐derived cytokines such as IL‐6 and IL‐4 can influence the glycosylation machinery of IgA‐producing B cells. Dysregulated cytokine environments within GCs may alter the expression of glycosyltransferases responsible for IgA1 O‐glycosylation, thereby facilitating the generation of Gd‐IgA1 [46, 54]. The skewed expansion of Tfh2 and Tfh17 subsets reported in patients with HSP may further enhance IgA production, with Tfh17 cells being particularly efficient at promoting IgA class switching [12].

Further supporting the role of Tfh cells in IgA dysregulation, Wang et al. [14] reported higher levels of Gd‐IgA1 in HSP patients’ blood, which were favorably linked with circulating CD4^+^ CXCR5^+^ ICOS^+^ Tfh cells, in addition to blood levels of IL‐6 and IL‐21. In another study on α‐1,4‐d‐polygalactosamine (PGA) antibodies in HSP patients by Meihua et al. [55], they found that serum levels of PGA‐IgA, CD4^+^ CXCR5^+^ Tfh cell percentage, and CD4^+^ CXCR5^+^ ICOS^+^ Tfh cell percentage were greater among HSP sufferers. PGA‐IgA levels were positively connected with Tfh cell percentage, particularly with CD4^+^ CXCR5^+^ ICOS^+^ Tfh cells, indicating that a rise in the proportion of CD4^+^ CXCR5^+^ ICOS^+^ Tfh cells could be the source of the increase in PGA‐IgA antibodies [55].

Consistent with these mechanistic insights, clinical studies have demonstrated significant alterations in B‐cell subpopulations in patients with HSP. One study reported increased proportions and absolute counts of total peripheral blood B cells, whereas plasma cell proportions were reduced. The proportion of naïve B cells was sharply reduced, but their absolute count was enlarged. The absolute count and proportion of nonswitched memory B lymphocytes rose, while no striking differences were observed within the proportion and actual number of switched memory B lymphocytes. Meanwhile, the proportion of CD4^+^ CXCR5^+^ Tfh cells within total lymphocytes raised greatly, which positively correlated with the proportions of total B lymphocytes, naïve B lymphocytes, nonswitched memory B lymphocytes, and switched memory B lymphocytes, but not plasma cells [56].

**Figure 1:**
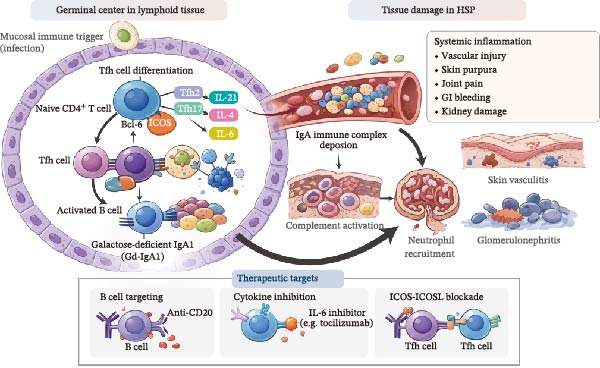
Tfh cells drive B‐cell activation and IgA production in Henoch‐Schönlein purpura (HSP) via IL‐21, IL‐4, and IL‐6, promoting immune complex formation and small‐vessel inflammation.

Taken together, these findings indicate that dysregulated Tfh–B‐cell interactions constitute an upstream immunological mechanism driving the activation, expansion, and class switching of autoreactive B cells. This process ultimately leads to abnormal IgA production, increased Gd‐IgA1 formation, and immune complex deposition, thereby accelerating the progression of HSP. This Tfh–B‐cell–driven pathogenic cascade, from Tfh‐cell differentiation and cytokine secretion to IgA immune complex deposition, complement activation, tissue injury, and related therapeutic targets, is illustrated in Figure 1.

### 4.3. IL‐6

Tfh cell–secreted IL‐6 has been shown to perform a crucial part in HSP pathogenesis. The level of IL‐6 in HSP patients is higher and strongly linked with the amount of CD4^+^ CXCR5^+^ ICOS^+^ Tfh cells [14, 56–59]. In acute inflammation, IL‐6 activates endothelial cells, which triggers the release of monocyte chemotactic proteins, enhanced production of adhesion proteins, and neutrophil migration to the inflamed site. This process may potentially lead to endothelial cell damage [60]. Furthermore, in adult HSP patients, a strongly favorable correlation among IL‐6 and IgA antiphosphatidylserine‐prothrombin (PSPT) complex antibody levels was observed [28]. IgA anti‐PSPT is an antithrombinogen antibody that induces hypercoagulability of the blood, thereby promoting thrombosis [61]. Upregulated IgA anti‐PSPT has been detected in HSP patients, and there was a clear correlation among IgA anti‐PSPT and higher C‐reactive protein (CRP) values [62]. CRP is an inflammatory biomarker, and increased CRP may contribute to clinical deterioration [62]. These findings indicate the oversecreted IL‐6 in HSP patients may contribute to leukocytoclastic vasculitis by activating endothelial cells and neutrophils and promote coagulation via inducing IgA anti‐PSPT. At the same time, overexpressed IgA anti‐PSPT further leads to higher CRP levels, thus aggravating the inflammatory response in HSP patients. Additionally, markedly increased IL‐6 and IgM anti‐PSPT in HSP patients with gastrointestinal symptoms were observed compared to those without such symptoms [28]. A research on acute mesenteric ischemia found a substantial increase in blood levels of IL‐6 in clients, suggesting a potential link between gastrointestinal involvement in HSP and abnormal augmentation of IL‐6 [28].

Besides the above, IL‐6 has been found to be closely associated with kidney damage in HSPN. Data show that the HSPN group produces significantly more IL‐6 than the simple HSP category, abdominal HSP category, and healthy control category, with IL‐6 levels in HSPN patients positively related with the degree of pathological grading [63]. Another research discovered a link between children’s HSPN and IL‐6 excretion in the urine, with HSPN patients having significantly higher IL‐6 concentrations in their urine relative to both normal controls and HSP patients without nephritis [64]. Therefore, IL‐6 levels may act as a potential indicator for distinguishing HSP with or without nephritis. As for the potential mechanisms by which IL‐6 promotes kidney injury, Sugiyama et al. [65] found profoundly elevated serum IL‐6 levels in HSPN patients compared to IgA nephropathy (IgAN) patients and a significantly positive connection among IL‐6 and blood Gd‐IgA1 enrichments. In HSPN patients, they also confirmed a significantly beneficial relationship among the glomerular meniscus and blood Gd‐IgA1. As previously discussed, the build‐up of Gd‐IgA1 immunity complexes within the kidneys induces renal damage, including glomerulosclerosis and interstitial fibrosis. Therefore, IL‐6 may contribute to the advancement of nephritis in HSPN patients through their association with Gd‐IgA1.

### 4.4. IL‐4

Several studies have shown that IL‐4 levels are abnormally elevated in patients with HSP [25, 43]. Increased IL‐4, as a primary effector of Th2‐type immune responses, may drive the development of naïve T cells towards Th2 cells, leading to a Th1/Th2 imbalance. Th2‐type immune reactions cause a release of a large amount of IL‐4, which causes aberrant proliferation and stimulation of B cells, resulting in excessive Ig synthesis as well as the onset of HSP.

## 5. Mechanisms of Abnormal Tfh Cell Expression in Patients With HSP

Wang et al. [66] have proposed a possible mechanism to explain the overproduction of Tfh cells in patients with HSP. According to their research, Tfh‐related regulatory factors were found to be abnormally expressed in these patients. Bcl‐6 and c‐MAF mRNA levels within circulating CD4^+^ T lymphocytes rose, but B lymphocyte–induced maturation protein 1 (Blimp‐1) mRNA levels dropped. Bcl‐6 is a transcription element that regulates Tfh cell development. CD4^+^ lymphocytes lacking Bcl‐6 cannot develop towards Tfh cells, with c‐MAF enhancing IL‐21 and CXCR5 production. The Bcl‐6 and c‐MAF combination can activate CXCR5, PD‐1, and ICOS, boosting Tfh development and functional differentiation [67, 68]. Blimp‐1, as an antagonistic factor of Bcl‐6, predominantly inhibits Tfh differentiation and development. Blimp‐1 can directly restrain the expression of Bcl‐6, and its activity can also be hindered by Bcl‐6[69]. Tfh cell overexpression in HSP patients may be related to the aberrant regulation of these factors.

## 6. Distinct Roles of Tfh Cells in HSP and IgAN

Although HSP and IgAN share several immunopathological features, emerging evidence points to notable differences in their immune regulation. HSP manifests as a systemic vasculitis, whereas IgAN primarily affects the kidney. In HSP, cTfh cells are markedly elevated and closely correlate with disease activity and serum IgA levels [43], whereas Tfh cell alterations in IgAN are generally more subtle and appear largely confined to mucosal immune compartments. This distinction reflects broader differences in the nature and extent of immune activation between the two conditions.

The immune environment in HSP also tends to be Th2‐biased, with increased IL‐4 production and expansion of Tfh2 subsets [70], which may facilitate systemic B‐cell activation and promote widespread deposition of IgA immune complexes in small vessels. Although mucosal immune dysregulation contributes to both diseases, the underlying mechanisms diverge: IgAN is strongly associated with aberrant immune responses in the GALT and tonsils [11], whereas HSP involves systemic immune activation—often triggered by infections—that leads to widespread immune complex deposition and vascular inflammation.

Collectively, these observations suggest that while HSP and IgAN share common pathogenic pathways, systemic Tfh‐mediated immune activation plays a more prominent role in the pathogenesis of HSP.

## 7. Comparison of Tfh Cell Dysregulation in HSP and SLE

Tfh cells are also critically involved in the pathogenesis of SLE, another autoimmune disease characterized by abnormal B‐cell activation and autoantibody production [71]. However, the functional characteristics of Tfh cells differ between HSP and SLE.

In SLE, expansion of Tfh cells leads to excessive GC reactions and the production of multiple pathogenic autoantibodies, including antidouble‐stranded DNA antibodies. Key regulatory pathways such as ICOS signaling, Bcl‐6 expression, and IL‐21 production play central roles in sustaining Tfh cell responses in SLE [13, 71].

In contrast, HSP is characterized by a predominantly IgA‐biased humoral immune response. Tfh cells in HSP primarily promote IgA production rather than the broad spectrum of autoantibodies observed in SLE [1]. Furthermore, HSP is frequently associated with infection‐related triggers and mucosal immune activation, which may influence Tfh cell differentiation through cytokines such as IL‐6 and IL‐4 [6].

These differences highlight how similar Tfh‐mediated immune pathways may lead to distinct autoimmune diseases depending on the immunological context.

## 8. Therapeutic Strategies Targeting the Tfh–B‐Cell Axis

Given the central role of Tfh cells in promoting autoreactive B‐cell responses and abnormal IgA production, targeting the Tfh–B‐cell axis has emerged as a promising therapeutic strategy for IgA‐mediated diseases, including HSP.

B‐cell depletion therapy represents one of the most direct approaches to interrupt the interaction between Tfh cells and B cells. Rituximab, an anti‐CD20 monoclonal antibody, has been used in patients with severe or refractory HSP and HSPN. Clinical reports have shown that rituximab treatment can reduce proteinuria and improve disease activity, supporting the critical role of B cells in the pathogenesis of IgA‐mediated diseases [72].

Cytokine‐targeted therapies may also modulate Tfh cell differentiation and function. IL‐6 is an important cytokine involved in Tfh differentiation through the activation of the STAT3 signaling pathway. Blocking IL‐6 signaling with monoclonal antibodies such as tocilizumab has shown therapeutic efficacy in several autoimmune diseases and may indirectly suppress Tfh‐mediated B‐cell responses [29]. In addition, IL‐21, a key cytokine produced by Tfh cells, promotes B‐cell proliferation, plasma‐cell differentiation, and Ig class switching. Experimental studies have demonstrated that inhibition of IL‐21 signaling can significantly reduce plasmablast differentiation and antibody production, highlighting the potential of targeting this pathway to regulate Tfh‐driven humoral immunity [73].

Beyond cytokine pathways, costimulatory signals are essential for Tfh cell development and GC formation. The ICOS–ICOSL signaling pathway plays a central role in maintaining Tfh cell function and B‐cell help. Experimental studies have shown that blocking ICOS signaling can reduce Tfh cell numbers and impair GC responses, suggesting that this pathway represents a potential therapeutic target for diseases characterized by abnormal Tfh activation [10].

More recently, therapies targeting B‐cell survival factors, particularly the BAFF/APRIL pathway, have attracted increasing attention in IgA‐mediated diseases. Agents that inhibit these pathways can reduce B‐cell activation and pathogenic IgA production and are currently under investigation in clinical trials for IgAN. Because these pathways regulate B‐cell maturation and antibody production, they may also indirectly regulate B‐cell activation and IgA production pathways, which are closely linked to Tfh cell function [74].

Collectively, these therapeutic strategies targeting different components of the Tfh–B‐cell axis, including B‐cell depletion, cytokine blockade, costimulatory pathway inhibition, and modulation of B‐cell survival factors, may represent promising approaches for the treatment of HSP. However, further studies are required to determine their efficacy and safety specifically in HSP.

## 9. Conclusions

HSP is a disease with a complex etiology. The function of T cells in the pathological process of HSP has grown into a major study issue in recent years. The involvement of Tfh cells within HSP is yet unknown as a subtype of T cells, as there are significant discrepancies between different study findings. Most studies, for example, demonstrate an increase in the number of circulating CD4^+^ CXCR5^+^ Tfh cells among HSP patients [14, 56–59]. However, Audemard‐Verger et al. [75] discovered that in HSP individuals, the fraction of CD4^+^ CXCR5^+^ Tfh cells among circulating memory CD4^+^ T lymphocytes was reduced, while CXCL13 production in the plasma was elevated. Tfh cells expressing CXCR5 move to lymph nodes under the chemotactic impact of CXCL13, allowing them to localize in the B cell zone of GC and interact with B cells [14]. CXCL13 increase may confine circulating CD4^+^ CXCR5^+^ Tfh cells into secondary lymphoid organs, causing them to participate in IgA synthesis and, as a result, reduce CD4^+^ CXCR5^+^ Tfh cells within the blood [76]. Similarly, different studies have produced distinct results regarding CD4^+^ CXCR5^+^ PD‐1^+^ Tfh cells. The majority of research results show no significant variations within the number of circulating CD4^+^ CXCR5^+^ PD‐1^+^ Tfh cells among HSP patients and normal controls, and the amount of CD4^+^ CXCR5^+^ PD‐1^+^ Tfh cells is not statistically connected with blood IgA, IL‐6, or IL‐21 levels [56, 57, 59]. A research, however, found that individuals with HSPN had considerably more peripheral CD4^+^ CXCR5^+^ PD‐1^+^ Tfh cells than normal controls. The frequency of CD4^+^ CXCR5^+^ PD‐1^+^ Tfh cells dropped after therapy, indicating that those cells are implicated in the pathogenesis of HSPN [58]. Similar findings were obtained throughout a research investigating the treatment of HSPN with total glucosides of paeonia: Circulating CD4^+^ CXCR5^+^ PD‐1^+^ Tfh cells rose in patients, as did IL‐4 and IL‐21 levels in the blood. The increases of CD4^+^ CXCR5^+^ PD‐1^+^ Tfh cells, IL‐21, and IL‐4 all appeared to be strongly correlated with each other [25]. Furthermore, multiple investigations have produced conflicting results regarding the fraction of CD4^+^ CXCR5^+^ ICOS^+^ PD‐1^+^ Tfh cells in HSP patients. Zhang et al. [58] found little variation within the percentage of CD4^+^ CXCR5^+^ ICOS^+^ PD‐1^+^ Tfh cells among patients with purpuric nephritis and healthy controls, while Liu et al. [59] found that circulating CD4^+^ CXCR5^+^ ICOS^+^ PD‐1^+^ Tfh cells seemed broadly higher in every group of HSP patients, especially in mixed‐type patients. It is well assumed that the PD‐1 molecule can negatively influence T‐lymphocyte activation and B‐lymphocyte activity within GC [76, 77]. The differences found in these investigations indicate that the function of PD‐1–expressing Tfh cells within HSP development needs further exploration. Table 1 shows the expression of Tfh cells and their cytokines in various investigations.

**Table 1:** Abnormal Tfh cells and their cytokines in HSP.

Source	Findings	Ref.
Children	CD4^+^ CXCR5^+^ ICOS^+^ Tfh cells, IL‐21, and IL‐6 increased in HSP patients	[14]
	CD4^+^ CXCR5^+^ PD‐1^+^ Tfh cells, IL‐21, and IL‐4 increased in HSP patients	[25]
	Tfh2, Tfh17, and IL‐4 increased in all types of HSP patients, but Tfh1 decreased in abdominal HSP patients	[43]
	CD4^+^ CXCR5^+^ Tfh cells increased in HSP patients	[56]
	CD4^+^ CXCR5^+^ and CD4^+^ CXCR5^+^ ICOS^+^ Tfh cells increased in HSP patients	[55]
	CD4^+^ CXCR5^+^, CD4^+^ CXCR5^+^ ICOS^+^ Tfh cells, IL‐21, and IL‐6 increased in HSP patients	[57]
	CD4^+^ CXCR5^+^ ICOS^+^, CD4^+^ CXCR5^+^ ICOS^+^ PD‐1^+^ Tfh cells, and IL‐21 increased in HSP patients	[59]
	CD4^+^ CXCR5^+^ ICOS^+^ Tfh cells increased in HSP patients	[66]
Adults	CD4^+^ CXCR5^+^, CD4^+^ CXCR5^+^ ICOS^+^, CD4^+^ CXCR5^+^ PD‐1^+^ Tfh cells, and IL‐21 increased in HSP patients	[58]
	CD4^+^ CXCR5^+^ Tfh cells decreased in HSP patients	[75]

While HSP can be self‐limiting to a certain extent, it poses a significant threat to several organs, gravely impacting the patient’s well‐being and may even result in fatality. Clarifying the role of Tfh cells in HSP may provide important insights into the disease mechanisms and reveal potential therapeutic targets. Future studies integrating clinical investigations and experimental models will be necessary to further elucidate how dysregulated Tfh–B‐cell interactions contribute to abnormal IgA production and systemic vasculitis.

## Funding

This work was supported by the National Natural Science Foundation of China (Grant 81872533), the Hunan Provincial Natural Science Foundation of China (Grant 2019JJ40427), and the Changsha Municipal Natural Science Foundation (Grant kq2502004).

## Conflicts of Interest

The authors declare no conflicts of interest.

## Data Availability

Data sharing is not applicable to this article as no datasets were generated or analyzed during the current study.
